# Beyond trust: Amplifying unheard voices on concerns about harm resulting from health data-sharing

**DOI:** 10.1177/23992026211048421

**Published:** 2021-10-01

**Authors:** Stephanie Mulrine, Mwenza Blell, Madeleine Murtagh

**Affiliations:** 1 Department of Social Work, Education & Community Wellbeing, Northumbria University, Newcastle upon Tyne, UK; 2 School of Geography, Politics, and Sociology, Newcastle University, Newcastle upon Tyne, UK; 3 School of Social & Political Sciences, University of Glasgow, Glasgow, UK

**Keywords:** Data, data-sharing, underrepresented groups, qualitative methods

## Abstract

**Background:**

The point of care in many health systems is increasingly a point of health data generation, data which may be shared and used in a variety of ways by a range of different actors.

**Aim:**

We set out to gather data about the perspectives on health data-sharing of people living in North East England who have been underrepresented within other public engagement activities and who are marginalized in society.

**Methods:**

Multi-site ethnographic fieldwork was carried out in the Teesside region of England over a 6-month period in 2019 as part of a large-scale health data innovation program called Connected Health Cities. Organizations working with marginalized groups were contacted to recruit staff, volunteers, and beneficiaries for participation in qualitative research. The data gathered were analyzed thematically and vignettes constructed to illustrate findings.

**Results:**

Previous encounters with health and social care professionals and the broader socio-political contexts of people’s lives shape the perspectives of people from marginalized groups about sharing of data from their health records. While many would welcome improved care, the risks to people with socially produced vulnerabilities must be appreciated by those advocating systems that share data for personalized medicine or other forms of data-driven care.

**Conclusion:**

Forms of innovation in medicine which rely on greater data-sharing may present risks to groups and individuals with existing vulnerabilities, and advocates of these innovations should address the lack of trustworthiness of those receiving data before asking that people trust new systems to provide health benefits.

## Introduction

In most health systems at present, the point of care is also a point at which health data are generated. In some cases, healthcare practitioners are being asked to act as points of contact for the consent or opt out process for data-sharing for direct clinical care, research purposes, or some combination of the two. Some health data are legally considered personal data under General Data Protection Regulation (GDPR), whereas other data are to be used in anonymised form, making them not legally personal data. However, some sensitivities may remain about the uses of those data because these uses may have implications for patients, families, or whole groups of which they are a part (e.g. ethnic groups, gender or sexual minorities, or other groups identified and targeted for public health intervention).^1^

In England, the National Health Service (NHS England) holds personal data about more than 55 million people in electronic health records of one form or another, although there is no single integrated system at present. The volume of data increases daily because the service deals with over 1 million patients every 36 hours,^2^ and increasingly this includes genomic data.

NHS data have begun to be discussed as representing a “goldmine”^3^ variously valued at GB£9.6 billion annually^4^ (over US$12 billion). In the implementation of “care.data” (2014), plans to share and sell NHS data were met with a strongly negative public response and ensuing media scandal.^5^ Nonetheless, data have continued to be sold^6,7^ or given away,^8^ but NHS England itself does not widely publicize this. Advocates of health data–related innovation have become more vocal, deploying normative arguments—from the relatively benign but reductive #datasaveslives Twitter campaign starting in 2018, to articles claiming an unwillingness to share health data for research and innovation is actively harmful, responsible for thousands of deaths and billions of pounds of unnecessary tax expenditure^9,10^—which mask the privilege inherent in such arguments. These echo other claims that there is a moral obligation to participate in health research.^11–14^ More recently, the Department of Health and Social Care’s 2021 Guidance entitled “A guide to good practice for digital and data-driven health technologies,” an update to the (also voluntary) 2018 “Code of Conduct for Data-Driven Health and Care Technologies,” talks about adopting data-driven technologies “responsibly and in a way that is conducive to public trust.”^15^ Yet contemporaneously, NHS data-sharing initiatives have received yet more negative public attention, leading to legal challenges mounted on behalf of the public in the courts both because of a lack of transparency and serious concerns about ethics.^16–22^

Alongside this, there are serious ongoing difficulties with de-identification of personal data^23–25^ and a growing list of harms related to uses of personal data internationally.^26^ In particular, there is growing evidence of a patterning to data-related harms, such that certain groups (e.g. people living in poverty, trans people, and ethnic minorities) face particular threats from datafication and data-sharing within administrative, health, welfare, and/or social care systems, including, but not limited to, intensification of discrimination by various means.^27–29^

Research demonstrates that poorer health is often associated with membership of these same groups, often denoted “hard to reach” by health researchers; among these, some well-researched examples would be ethnic minority groups.^30^ In particular, ethnic minority underrepresentation in health research has been traced to concerns within particular groups about stigma at individual and group levels (including concerns about the way group-level findings are reported) and expectations of a lack of shared benefit of health research (a belief that benefits will only accrue to white populations, including researchers, regardless of how inclusive the data) (see George et al. 2014 for a review).^31^ A study of ethnic minority students demonstrated that, contrary to assumptions, increasing knowledge about genetic research increased concerns about negative impacts, including privacy violations, discrimination, and eugenic consequences,^32^ putting to rest assumptions that concerns are based on ignorance. While more representative data will prevent some biased outcomes (e.g. AI’s inability to recognize melanomas on dark skin) others cannot be so resolved (e.g. biases embedded in data sets through discrimination or unequal access to healthcare).^27,33^ There is a growing recognition that there are ethical issues, issues which cannot be reduced to the results of underrepresentation, which need special scrutiny where the personal data of “vulnerable groups and individuals” is used, for example, use of ethnic minority groups’ genotype and/or phenotype data.^34,35^

We argue that it is important to engage those groups with specific concerns and at particular risk of harm. Large-scale studies that purport to capture and report majority views^36,37^ do not suffice. Following Luna’s theorizing of vulnerability emphasizing its complex, contextual nature, we look to avoid stereotyping and recognize that existing vulnerabilities, often produced by marginalization in society, can lead to cascading effects of great harm if they meet triggering conditions (e.g. someone with reduced immune function without access to medical care may be more likely to die when there is an outbreak of infectious disease because they are more likely to become infected and less able to get treatment).^38^ As a result, we set out to engage with groups with socially produced vulnerabilities. Our identification of groups was informed by previous qualitative work undertaken by the authors in the same region, which identified public concern and disquiet over those underrepresented, some of whom were felt to be vulnerable to data-related harms.

## Methods

We present qualitative findings from multi-site ethnographic research carried out between March and September 2019 in the industrial conurbation of Teesside, North East England. This research was carried out as part of Connected Health Cities North East and North Cumbria (CHC NENC)’s program of public engagement activities. Ethnographic research methods go beyond standard engagement practices, which aim to elicit public views, by going to participant groups rather than summoning them to the research or engagement practice (e.g. surveys and consultations). Participant observation, a long-established ethnographic method, allows the researcher to develop an intuitive and contextualized understanding of the perspectives of members of a group in their own setting and to reduce the extent to which that behavior is influenced by the fact that the group is under study.^39^

Ethical approval was received from the Newcastle University Ethics Committee (10275/2018) before commencement of fieldwork. M.B. and S.M. identified voluntary and community sector organizations working with marginalized groups via Internet searches and their networks. Contact was made by email or by attending publicly advertised drop-in sessions or events to introduce SM, the research associate on the project, a female researcher with a PhD in Sociology, and the research project itself. The research project was explained, information sheets were offered, and people were given the chance to ask questions. The information sheet was developed based on recommendations from a trained speech and language therapist in such a way as to be easy to read, in order to be appropriate for those with low literacy. Following this, S.M. sought verbal or written informed consent to speak to the staff or volunteers or to attend events in order to make contact with people from the organizations’ target groups in order to invite them to participate in the research. Participants were made aware that SM was acting as an independent researcher not involved in the development of health data-sharing policies or technologies, that the research followed previous work to understand public perceptions of health data-sharing, and that it aimed to broaden the insight of a range of particular groups. Members of the target groups and those people who worked in the organizations comprised the research participants. All participants were required to be over the age of 18 in order to participate in the study and verbal informed consent was sought from all participants and some participants also gave written consent using a consent form. Where participants had limited English, careful verbal explanations in English were supplemented by the use of trusted simultaneous interpreters using languages in which they were more proficient. The requirement for subjects or their legally authorized representatives to provide written informed consent prior to study initiation was waived by our Institutional Ethics Committee. The reasons for this requirement being waived were (1) the fact we used a methodological approach (ethnographic research) that utilizes all interactions as informative makes consent only meaningful as an ongoing verbal ethical contract and (2) in order to be flexible to the literacy needs of all those potentially participating, that is, to not require people to sign things they cannot read as this is both unfair and potentially harmful, since not all people involved had literacy in any language. This verbal consent was not recorded on any formal paperwork but no fieldnotes were written about conversations where there was no verbal consent to participate.

As a participant observer, SM took part in a range of different formal and informal activities attended by members of the groups, carrying out repeated convenience sampling within this purposively sampled local context. This approach also helped her to gain trust and access to honest and nuanced reflections on sensitive issues related to the topic. S.M. compiled ethnographic fieldwork record—systematic records of day-to-day interactions, observations, and informal conversations. By building rapport during informal encounters, research participants were able to think about the relatively unfamiliar subject of health and genomic data-sharing.

Ten community and voluntary sector organizations took part. They varied in their focus, size, and demographic spread. To maintain confidentiality, participating organizations are not named. The social issues that they focused on included:

- Poverty: working with and for communities to campaign for change at a local and national level.
- Substance misuse: providing mentoring and support for men to overcome addiction, mental health issues, and unemployment.
- LGBT issues: providing safe spaces for members of the LGBT community to meet and undertake creative projects with equality at heart.
- Asylum seekers: working with refugees and asylum seekers to better understand the specific challenges they face.
- Domestic abuse: providing accommodation, counseling, and support for women leaving abusive relationships.
- Social isolation: a range of community projects aimed at reducing loneliness and increase well-being.
- Food poverty: giving those in crisis access to basic necessities and help to improve their situation.

Other organizations were approached, but many community organizations were overwhelmed by the challenge of performing their core activities without adequate funding, so participating in research was not a high priority, even if the topic was important to them.

## Data analysis

Analysis was thematic and primarily inductive, based on the principles of grounded theory.^40^ Two authors (M.B. and S.M.) engaged in close reading of the fieldnotes and discussions of each field visit, during and following data collection. Key themes, patterns, and variation^41^ were noted and developed.^42^ Interpretations were then tested and challenged in discussion with the third author (M.M.) and refined. This analysis formed the basis of synthetic narrative accounts (the vignettes), thereby ensuring no single participant is recognizable on the basis of the presented findings. Vignettes were assessed for conceptual coherence and sense making, including testing, challenging, and refining initial interpretations, through discussion and critical reflection by all authors (interpretive validity).^41,42^ Each vignette combined elements of details of the accounts of different participants in order to protect each person’s identity.^43^ In accordance with the conditions of ethical approval, no real names of individuals or institutions are included, and identifying information has been excluded. The names used in the text are made up and do not belong to any participant in the study but cannot be considered pseudonyms since no one individual’s story is being reflected across any whole vignette. Access to these data as supplementary materials or via third-party repositories is not allowed under ethical approval, these data are highly sensitive and identifiable. The vignettes function like case studies for the reader, enabling them to see the links between the contexts of people’s lives and their concerns about health data being shared. We do this in an attempt to respond to Dyer and Wilkins’s call to create “more persuasive and memorable” stories which have the potential to offer an “a ha” experience for the reader through rich descriptions.^44^ Furthermore, the vignettes are written in a style that reflects the voices of those whose stories they embody. The results presented do not claim to be an exhaustive list of potential risks of harms but rather to surface risks which have, for these participants, been hitherto obscured. This research did not aim to reach saturation. Following Saunders, we argue that saturation is a concept that is not easily defined nor consistently applied. In the context of ethnographic approaches that utilize an inductive approach, the notion of narrative accounts of our participants, we are careful not to treat these as being “complete” but rather part of the continuous informing of research in this area.^45^ The insights we describe are informative and highlight substantial areas of concern for those at risk of harm from data-sharing. However, they do not constitute all the risks and harms that may occur or all themes which could be identified from the same data.

## Results

Our analysis identified three areas of concerns about sharing sensitive health data: data and information about *substance misuse*, *sexual health*, and *mental health*. We found that concerns about data-sharing are most acute for asylum seekers, those experiencing domestic violence, transgender people, offenders, and ex-offenders: those who are vulnerable to the greatest potential harms and discrimination from data-sharing are already those in society who are most marginalized and disadvantaged. Intersections of socially produced vulnerabilities create even more concern for people about their data. For example, sexual and reproductive health data for lesbian, gay, bisexual, and transgender (LGBT) asylum seekers can be particularly sensitive and sharing beyond the NHS can represent a threat to people’s lives. It is relevant to note that there are ongoing hate campaigns in social, online, and print media and scholarly publication in the United Kingdom and internationally about transgender people which involve sharing information about transgender people who have been convicted of crimes and detailing gender-related health information about them. In such a context, trans offenders’ health information becomes a tool to be used against the whole group, whether such uses can be considered research or not.^46,47^

Greg’s Story

Being an advocate for, and having expertise by way of experience of, LGBT issues, Greg is generally forthcoming in sharing details of his life experience. However, Greg is very aware that being LGBT, and particularly trans, can be viewed with prejudice. So many of those he supports wish to keep these personal details private. He is supportive of their preferences and has specific concerns. He is aware that lack of sensitivity by healthcare professionals can lead to traumatic events being inflicted or relived by using previous personal information that deliberately misgenders or mischaracterises their lived experience.

Box 1. Greg’s Story

Greg lives in a small village on Teesside. As a trans-man, he grew up feeling uncomfortable with his body and the fact people referred to him as a girl. When he was younger he struggled to talk with friends and family about his feelings and found it difficult to get information or support.

Now in his late 30s, Greg has managed to obtain gender-affirming surgery. It was not a quick or easy process, and he lost the support of many friends and family along the way. But Greg now had, what he and many others in the LGBTQ community refer to as, his chosen family. In addition, he had learned a great deal from his struggles to get appropriate care from the NHS.

Greg tries to be an ‘open book’. He wears the scars of his transition quite plainly on his body, with a large scar around his forearm which was the site of a skin graft. Occasionally people are inquisitive, and Greg is happy to share as he is a believer that knowledge will help tackle the ignorance, fear and prejudice that the LGBTQ community faces. Whilst he ‘is all for teachable moments’ he recently presented at A&E feeling very unwell. He was triaged by a nurse who was ‘very nice’ but seemed to get stuck on gathering details about the type of gender-affirming surgery he had received years before while ignoring his immediate symptoms. Given his acute symptoms and his panic, he felt this was not the appropriate time. While he tried to be polite and answer her questions, he felt woozy and rushed the conversation. He was suffering from norovirus, and after a brief admission to hospital, he made a full recovery. Yet this experience stayed with him.

Greg finds himself helping LGBTQ people from all walks of life navigate the NHS. He is aware that NHS resources are limited but he also helps other transgender people to understand and anticipate some of the more specific expectations of them, for instance, he advises people that getting a legal name change helps show doctors the strength of their intentions. These can be unsettling and anxious times and Greg has seen people experience hope upon hearing they will receive support and surgery via the NHS and heartbreak when they are told they are not yet eligible.

Greg is aware that the transgender community is relatively small and that many clinicians lack knowledge about transgender people. During a discussion at a weekly LGBT support group, he attends another attendee had been admitted to a local hospital for a catheter to be removed. “A nurse came into the cubicle and asked me to roll onto my side saying ‘We might as well check your prostate while we’re here’, I just laughed at her and said ‘I think you should check my medical notes’”. Everyone fell about laughing, but Greg was also a little worried about what this said about the assumptions that healthcare practitioners still had locally, and how routinely the notes provided were or weren’t thoroughly read.

That same day, another group member who had been attending off and on for many years appeared. She too had undergone a long journey to transition and had been post-operative for several years. Greg knew her well and was concerned to see her looking sad and agitated. He pulled her aside to a quiet area of the room to offer support and a cup of tea. Initially, it was difficult to get her to open up, but she described a recent trip to the doctor for something routine. Her usual GP, for who she had known for decades and throughout her transition, was on holiday and so she saw a different doctor. Without warning, during the consultation, the GP referred to the sex assigned to her at birth and, when she objected to this, he suggested she needed a referral to psychiatric services. She had been living as a woman for many years was surprised to be misgendered by a doctor caring for her and was troubled by his transphobic attitude. Having experienced bullying, harassment, and abuse for many years before receiving hormones and surgery, she felt she was suddenly plunged back into her painful history. Greg attempted to comfort her and offered to help her make a complaint to the doctor’s practice. She was still clearly shaken up by the experience and left saying she would give it some thought but that complaining might make her feel worse by having to describe the experience again.

After getting home that evening, Greg tried to relax, but he was frustrated and emotionally drained as hearing about others’ experiences reminded him of the highs and lows of his past and of how many transgender people still die by suicide.

The discriminatory treatment people from marginalized groups receive in the NHS is part of the backdrop for considering whether data-sharing and data-related innovation is something people feel can be trusted. From the experiences of our participants and based on prior research,^48–51^ NHS services are not yet able to handle such sensitive information without stigmatizing people: wider sharing amplifies this risk. Personal sensitive information, such as HIV status, is known to have been mistakenly shared by the NHS.^52^

Craig’s Story

As Craig finds himself in challenging circumstances whilst aiming for recovery, his concern and worry about how others may treat him in light of his substance misuse is profound. Based on previous experience wherein his access to care was made difficult due to discriminatory practices. He understandably feels apprehensive about a potential negative recurrence if he seeks help and the information held about him is used to stigmatize his condition. Feeling powerless to challenge or resist this instead he avoids interactions with healthcare professionals.

Box 2. Craig’s Story

Craig has been unemployed for several months. He is 39 and has lived in Teesside all his life. Often in and out of work, he finds the churn of precarious work, welfare, and his addiction has taken a toll on his physical and mental health.

Having split up from his partner due to his financial, substance abuse, and mood problems, Craig does not see his two children as much as he would like. When he does see them, it is under a supervision order. This situation makes him feel under pressure and as though he is constantly being scrutinized.

Years ago, Craig stole to feed his drug addiction and ended up in prison. He has since attempted a methadone program which helps keep the worst impulses at bay. However, his recent financial and family troubles have led to an increase in his dependency on alcohol. While he knows his addictions need to be addressed for him to make positive steps forward in his life, it is difficult.

Craig’s dependency is compounded by where he lives, a place known for its high rates of crime. He finds it difficult to avoid the drug dealers he used to visit frequently. Whilst he has a group of friends, they are all also dealing with addiction.

Recently, Craig has made contact with a local charity that aims to support men who are facing addiction, homelessness, and unemployment. He feels encouraged because the first time he spoke with a volunteer, Julie, she suggested that the charity would be able to help him find more suitable and safe accommodation. They also said that they could help him detox safely and find a residential place at a rehab center. Craig is reluctant about entering rehab and hopes not to have to take such a drastic measure for two reasons. First and foremost, he would not want to be away from his children for a prolonged period of time and feels the support and proximity of his family—although also a pressure—would be important for his recovery. Second, the charity is new to him, and he is still trying to establish trust. The charity has a religious motivation and while they do not preach to him he is still a little cautious after mixed experience with other authority figures.

Julie has suggested that he visits his local general practitioner (GP). She has raised concerns about how unwell he looks and suggest that the GP could help with practical steps to recovery. The first time she mentioned it, Craig rejected this option and abruptly shut down the conversation. He knew she meant well but felt she did not understand.

Craig has had numerous interactions with health practitioners over the years. Many of these have been negative and at times significantly worsened his situation. He feels that when they learn from his notes that he is on a methadone program, they write him off as an addict; someone to be wary of and who should be treated differently.

After being admitted to the Accident and Emergency department (A&E) of a local hospital, a doctor there suggested that, as his illness was “self-inflicted”, he should not be given treatment. Craig responded angrily, and even though he felt he was not being aggressive and was only trying to defend himself, the police were called and the situation escalated. He felt like it was them against him, yet he felt powerless and unwell so just had to accept it. Information was being shared about him, and he was unable to express himself. This led to Craig feeling distrustful and even sometimes paranoid when it came to the idea of accessing healthcare again.

While Craig knows that there are particular steps he is expected to take in order to become well, his previous bad experiences mean that he prefers to try to do things himself rather than risk the judgment, and shame, and stigma of those who share information about him and may dictate aspects of his life, such as access to his children, his accommodation, and healthcare. Craig continues to be cautious about any possibility that information about him will be shared.

Data-sharing is a double-edged sword for vulnerable groups who feel they might potentially benefit from high-tech care, health research, or swifter information sharing among relevant direct care providers but suffer potential harms from that data-sharing. Indeed, it is also the case that for those moving home or region at short notice (e.g. to escape domestic violence) speedy electronic transfer of care records to new providers would reduce risk of harm—perpetrators of violence have accessed information to track down their targets through health service text messages.

Gemma’s Story

The crisis in which Gemma and her children find themselves demonstrates an intense, yet unfortunately common, need to find safety by leaving an established and settled life. In order to seek refuge she had to trust the support workers, but also felt a heightened sense of alert due to being made aware of the ways in which her abusive partner may attempting to locate her and her children by utilizing information about them. What had once seemed perfectly normal, such as a text reminder of a GPs appointment, now posed a potential threat.

Box 3. Gemma’s Story

Gemma is 29 years old, from Kent, and has two children: Maisie who is 12 years old and Dylan who is 8 years old. As a mother, Gemma has always tried to do what is best for her children; however, it has not always been easy. Maisie used to love unicorns and Disney films, but seems to think that is just for kids now and spends a lot of time on Instagram, which worries Gemma. Dylan requires a huge amount of time and attention as he has behavioral issues. Gemma has taken him to the doctor to discuss this frequently, but can feel nervous about being judged as a bad parent. ADHD and autism have been mentioned and, while she is relieved that she is being taken seriously, she is concerned about the effects of his new medication on his long-term health.

All of Gemma’s worries have been amplified and eclipsed in recent weeks. After being with Dylan’s father, Jack, for 9 years on and off, she and the children have finally left the family home. Jack had always been protective and sometimes jealous, but in the past 4 years, his behavior had become more and more agitated. At several points, she had sent the kids to stay with her sister for the night, and on more than one occasion she had moved herself and the kids in with her mother for a week or so.

Often these situations would improve for a short time, but it seemed that it was easy for Jack to blow up into a rage over the most trivial of matters. As he brought the majority of the money into the household, it had initially seemed fair to Gemma that he look after the finances. But he would scrutinize receipts and had scared the kids on one occasion in particular when Gemma had bought two pizzas that had been on sale but were not on the shopping list.

As time went on Gemma found herself making excuses for Jack more and more. But she had a shock, and found it initially difficult to deal with, when social services became involved due to concerns that Dylan’s schoolteacher had raised about her partner’s controlling behavior and bad temper. Despite Gemma’s best efforts the involvement of social services seemed to agitate Jack even more. She did her best to tip-toe around him, but one evening, he went into a rage and physically assaulted Gemma. It was not the first time. A concerned neighbor rang the police, but by the time they came, Jack was long-gone. In a daze, Gemma did not want things to be made worse, but she felt more unsafe and scared for her life than she ever had before. One of the police officers suggested they call a local women’s refuge, it felt like this time she had no choice and that it was now or never.

Gemma stayed there for a week with Maisie and Dylan, until Jack found out where the refuge was. A friend contacted her to say that he’d spoken with Jack, had tried to calm him down, but that he had stormed off making very unsettling threats about Gemma and the kids. The support workers at the refuge acted fast when Gemma told them he had found out where the refuge was. Within 20 minutes both she and the kids were in a car with their few belongings and were being driven out of the town she knew and grew up in. On their way, they were informed that due to demand, the nearest refuge with a space that day was in Haltborough in North East England. Gemma had never even heard of the place.

The first few days were incredibly stressful, the support workers were helpful, but even they were frustrated with the emerging situation. Dylan’s behavior was erratic, aggressive, and highly anxious. Within a couple of days, the medication that Dylan was on to help manage his behavior ran out. He started to lash out at Gemma, Maisie, and the refuge’s support workers when they tried to help or intervene. Many phone calls were made to her old GP, the new local GP, her old pharmacist, and the new local pharmacist, which seemed to leave her with conflicting instructions on how to get the particular medication Dylan was on. The support workers tried to help, but they constantly had to get approval from Gemma to speak on her behalf. Eventually after several attempts across 2 days, they discovered that the type of medication Dylan was taking was not available in the North East. Gemma nervously took Dylan to see a GP to explain the situation. She was relieved that the GP prescribed new medication for Dylan.

As Dylan’s behavior settled and Gemma was able to process the stress of the last days, weeks, and years, she realized that her life would never be the same again. The refuge was a great source of support and measures were being taken by the police against Jack. But Gemma worried a lot. She had been given a new phone at the refuge, an old-style handset that did not have the internet and could only make calls and send texts. This was because of the threat that based on Jack’s controlling behavior he might have cloned her phone. Gemma had never heard of this, and the support worker explained that ‘perpetrators’ (Gemma still found this language and label hard to process) could use software to illegally clone and access a victim’s phone without their knowledge. So, if she got a text from a friend to meet in a coffee shop, or a text reminder for an appointment with a doctor, a perpetrator could see it and then turn up and abuse, harass, or worse. All the worry about him finding out their location made Gemma struggle to trust, even people she previously would have done, with her sensitive information and data.

Participants wanted more information about how privacy violations or other harmful impacts of data and information sharing would be handled. Some asked pointedly who would decide what types of data were shared, with whom, and for what purposes. Many patients want more controls on how and with whom information is shared, rather than just an automatic wider sharing and use of their health data, this included a desire to have some control over the way the NHS contacts them.

Nasrin’s Story

Nasrin experienced multiple stressors whilst awaiting the outcome of her asylum claim in the UK which was compounded when she realised that information could be shared without consent between healthcare professionals, social workers and police if a safeguarding concern was raised. Attempting to get adequate information on her rights and protections was further frustrated by the highly changeable context in which she lives, where private contractors providing her accommodation are changed with little warning. Continuing instability leaves Nasrin and other asylum seekers with questions and confusion over what or who to trust in relation to their sensitive health information.

Box 4. Nasrin’s Story

Nasrin is a 20-year-old asylum seeker from Somalia. She came to the United Kingdom 2 years ago and hopes the Home Office will grant her refugee status and the right to remain. Nasrin lives in run-down accommodation provided for her, in the center of Shieldton, a mid-sized city in North East England. She has been placed in a house where she has to share a bedroom with a woman from Brazil with whom she does not share a language. They have different cultures and routines which cause tensions between them so Nasrin worries about getting up to pray at night because it disrupts her roommate and causes more friction, she worries the woman will complain about her to authorities.

Before coming to the United Kingdom, Nasrin lived near Mogadishu, but conflict in the area meant she feared for her life so she fled. She wanted to come to the United Kingdom and, in particular Shieldton, as that is where her mother had sought asylum. She hoped to be housed with her mother but unfortunately was not.

Whilst being reunited with her mother was wonderful, and she was glad to have her for support and guidance, there were many concerns and worries that seemed to mount up. This meant that sometimes Nasrin was extremely hesitant when situations arose that she felt could adversely affect her asylum claim.

Nasrin visits several different community drop-in sessions locally to chat with others and get advice. Both Nasrin and her mother find these invaluable, as they can access advice, information, and a space to improve their English skills. They have made friends and the sense of community is important for both of them, especially as they regularly witness and suffer xenophobic racist abuse in public spaces.

Recently Nasrin has been spending time with a new Somali friend, Ayanna, who described a recent prenatal check-up which greatly upset her. Ayanna who is in the third trimester of pregnancy was preparing to welcome her baby to the world. At Ayanna’s check-up, the midwife did a physical examination and realized that Ayanna had been subject to female genital mutilation (FGM). When Ayanna’s appointment finished, she walked the 15 minutes to her home. Upon arriving she found a police officer and social worker at her door. They explained that they had been instructed to contact her due to concerns from her midwife about the risk to her unborn child of FGM which is a crime in the United Kingdom. She explained to them that she was seeking asylum in the United Kingdom in part to escape the threat of FGM for her child. They left saying they would note what she had said.

Ayanna and Nasrin were surprised and worried. They had believed that in the UK discussions with healthcare professionals were confidential and yet somehow this information had been shared. As they did not fully understand why, they were concerned about seeking medical help in the future. Nasrin suggested to Ayanna that they speak with one of the drop-in volunteers for advice and to check if she should be worried about her asylum claim. However, the community outreach drop-in sessions have been busy due to rumors that all asylum seekers were going to be forced to move out of their properties at very short notice; “a matter of days”, some were saying. Lots of people had questions and were concerned.

Initially Nasrin was hopeful that the relocation might mean she got a room of her own, but then she became concerned that she might be moved far away from her mother and her friend. Ayanna had many concerns due to the fact she is heavily pregnant and has spent time and energy preparing her previously moldy accommodation for the arrival of the baby and felt worried about being moved somewhere which was more crowded or run-down.

The drop-in sessions felt more anxious than usual and Nasrin wanted to help her friend with her immediate relocation worries. They were advised that they could raise concerns via official routes and were given details of the Home Office to take the issue further. The volunteer was very supportive and helped provide information on what their rights were. However, they were still very nervous and worried that information about them might be shared and that if they expressed distress or unhappiness about the situation to a health worker, it might detrimentally affect their asylum claims.

## Discussion

As we can see from the above vignettes, some people have pre-existing deep-seated concerns about presenting to a doctor or allowing personal information to be shared. Eroding doctor–patient confidentiality, or even allowing any doubt that the information is not held in strict confidence, may push people away from accessing healthcare, creating both a public health issue and a social justice issue. There is already evidence that people avoid seeking important help as a result of the NHS’s recent history of sharing information with other organizations, with serious consequences for morbidity and mortality.^53^

During the period of fieldwork, NHS England announced its intention to share patient data with the Department for Work and Pensions (DWP). Participants expressed clearly and directly that they would stop accessing healthcare if they learn that their information might be shared with DWP. There is a fundamental lack of trust in the DWP to make fair decisions around ill health. Participants have no faith that an increase in data-sharing would change the DWP fundamentally, and indeed it would be naïve to think data-sharing would resolve structural and political issues. The many years of demonization of benefits claimants and negative rhetoric from politicians are clearly part of this context. Moreover, suggestions in the media that data science is being used to detect benefit fraud,^54^ despite high-profile work showing such systems are responsible for terrible outcomes for innocent people,^28^ do not encourage people to feel at ease.

This is not to say that people with vulnerabilities do not see the potential benefits to be had in terms of improved direct care. There are potential benefits to health data science, as well as day-to-day clinical practice of greater sharing of patient data. Yet, as other researchers have found, even those who have the most positive views of data-sharing do not want others to have unfettered access to data in their NHS records.^55^ Moreover, risks are not equally spread across society and we show vividly the reasons why some patients groups have particular concerns. Time and energy should be spent on addressing these broader contextual issues that generate eminently reasonable grounds for mistrust rather than exhorting patients to accept that their data should and will be shared, expecting them to trust clinicians and health data scientists. Arguably, the main limitation of our study is its lack of generalizability. Our selection of groups and individuals to approach in this study was not determined with the aim of generating data generalizable to any population but instead aimed to generate rich data suitable for reflecting concerns with enough context to be meaningful for readers who do not share the same socially produced vulnerabilities and to potentially surface concerns not readily identifiable in population-level research. It is interesting to note that in some cases, the staff or volunteers had concerns about members of their target groups participating in the research. They questioned whether people in difficult circumstances have more pressing issues to deal with in their lives. However, they agreed that the research was important and wanted to participate on behalf of these groups. It is worth thinking about just how and whether people in challenging social circumstances have the capacity to engage in more time-consuming and non-ethnographic public engagement work to talk about their perspectives and how this is likely to differ for the people with whom we engaged in this project. Our methodological approach and resulting vignettes offer a way to think about important aspects of people’s lives going forward in time. Dialogue allowed for discussions to avoid being about binary or dichotomous consents, which has been noted produces “an involuntary trust.”^55^ We have not tried to find out merely if some predetermined action is acceptable but have been open to listening to the voices of our participants in order to learn what is important to consider if data-sharing is to be scaled up for the delivery of data-driven health research and innovation. We suggest that where depth and communication of perspective are important, properly contextualized narratives, generated through in-depth qualitative research (e.g. ethnography), may be a useful tool for helping readers to gain understanding, rather than merely inventorying to produce a list of concerns.

Another limitation is that all participants were based in England, their views thus are more reflective of NHS England than healthcare in the United Kingdom as a whole since this is a devolved matter and thus run separately in Scotland, Wales, and Northern Ireland. Indeed, a recent high-profile data transfer plan was not intended to affect patients in Scotland, Wales, or Northern Ireland, only those in England, but did cause confusion because this was not always clear to the public.^56,57^ There is some reason to think patient views in these other countries may well be different, since, for example, data-sharing for research^58^ and care^59^ within GIG Cymru (the National Health Service within Wales) has developed in recent years without similar controversy.

Many participants asked questions about just how new data-sharing systems would work: how transparent processes for data-sharing and use would be; how people would be held to account in case of problems; and, indeed, how problems with new data-driven systems would be detected. We have given attention to these questions. Crucially, we have not assumed that it is lack of understanding driving the questioning. We, thus, explicitly reject the deficit model which remains embedded in much engagement work. In our view, these are questions about data governance and they are clearly important for the issue of ‘agreement’/consent to data-sharing. Yet, there are no clear answers to these in the United Kingdom at the time of writing. Thus, the deficit is in the information available to the public about how new data-sharing systems will work, and not a deficit in knowledge on the part of our participants. There may also be deficits in the methodological approach of those engaging with public attitudes to listen sufficiently to such concerns. Questions about mechanisms for accountability for protecting the privacy and the interests of the public are urgent. There has been a tendency in some other engagement work about data to present ethical concerns within constrained and largely hypothetically based trade-off scenarios where risks (privacy violation) must be considered alongside benefits (new drugs being developed),^60^ this does not allow a flexible engagement of the participants with the premises on which the trade-off is based. We see little value in such artificially constrained conversation. Moreover, public engagement work should not be so focused on majoritarian perspectives that it ignores the concerns about potential harms for vulnerable groups. Public engagement can be enriched by careful consideration of the wider context that surrounds the topic of interest and the wider lives of those with whom we seek to engage.

## Conclusion

Our overarching finding is that the context of past sensitive data handling by NHS England as well as the socio-political situation in the United Kingdom and globally with respect to the use and sharing of personal data are essential considerations for understanding people’s concerns about health and genomic data-sharing. This context does not create simple binaries of positive versus negative attitudes or easily quantifiable measures of acceptability in people’s minds. We have highlighted the importance of alignment between the intention to listen to unheard voices and the methods we have used to listen most effectively. The ethnographic approach we have used makes it possible to earn and, hopefully, deserve the trust of our participants as users of their personal data to develop understandings we can share with others. Based on our findings, we recommend that NHS data-sharing advocates focus efforts first on reforming existing systems of care and data management so that they are more respectful, person-centered, and safe for patients and second on the development and implementation of transparent and trustworthy data-sharing and data-driven technology governance that prevent harms, rather than advocating public trust.
